# Simultaneous Multi-Species Tracking in Live Cells with Quantum Dot Conjugates

**DOI:** 10.1371/journal.pone.0097671

**Published:** 2014-06-03

**Authors:** Mathias P. Clausen, Eva C. Arnspang, Byron Ballou, James E. Bear, B. Christoffer Lagerholm

**Affiliations:** 1 MEMPHYS – Center for Biomembrane Physics and Danish Molecular Biomedical Imaging Center (DaMBIC), University of Southern Denmark, Odense M, Denmark; 2 Molecular Biosensor and Imaging Center (MBIC), Mellon Institute, Carnegie Mellon University, Pittsburgh, Pennsylvania, United States of America; 3 Lineberger Comprehensive Cancer Center and Department of Cell and Developmental Biology, Howard Hughes Medical Institute, University of North Carolina, Chapel Hill, North Carolina, United States of America; J. Heyrovsky Institute of Physical Chemistry, Czech Republic

## Abstract

Quantum dots are available in a range of spectrally separated emission colors and with a range of water-stabilizing surface coatings that offers great flexibility for enabling bio-specificity. In this study, we have taken advantage of this flexibility to demonstrate that it is possible to perform a simultaneous investigation of the lateral dynamics in the plasma membrane of i) the transmembrane epidermal growth factor receptor, ii) the glucosylphospatidylinositol-anchored protein CD59, and iii) ganglioside G_M1_-cholera toxin subunit B clusters in a single cell. We show that a large number of the trajectories are longer than 50 steps, which we by simulations show to be sufficient for robust single trajectory analysis. This analysis shows that the populations of the diffusion coefficients are heterogeneously distributed for all three species, but differ between the different species. We further show that the heterogeneity is decreased upon treating the cells with methyl-β-cyclodextrin.

## Introduction

The technique of single particle tracking (SPT) offers, compared to other related optical microscopy methods (FRAP, FCS, STED, STORM etc.), the best combination of high spatial resolution (20–30 nm), fast temporal sampling (25 Hz–50 kHz), and a large field of view (typically >100 µm^2^) [1], [2]. The classical SPT probes are 40 nm diameter gold particles that are additionally stabilized and functionalized for specific molecular binding resulting in a hydrodynamic radii, *R_H_*, of ≥25 nm [1], [3]. These probes can be imaged at very high sampling frequencies (≤50 kHz) for very long periods of time (∼minutes) [3], [4], but their non-invasiveness in cells due to their large size remains a contested topic [1], [5]. Furthermore, as a consequence of the detection by Rayleigh scattering, high light intensity is needed, and multi-species SPT with gold particles is impractical.

SPT is also possible with fluorescent dyes and fluorescent proteins in which case SPT is sometimes alternatively called single-molecule fluorescence tracking (SMFT). But, the limited photo-stability of these probes most often results in only very short trajectories with a typical median length of 5–15 displacements [6], [7]. These trajectories are much too short for reliable analysis of single trajectories which has the consequence that the subsequent analysis in that case is most typically performed to yield an ensemble average for all molecules in both time and space [8]. Alternatively, statistical methods have been devised for analyzing the entire distribution of single trajectories [9] [10], e.g. to extract intracellular diffusive states and state transition rates from thousands of short single molecule trajectories [10]. In contrast, use of more photostable single molecule probes enables the collection of longer trajectories, which in turn allows for robust analysis and assessment of the individual single trajectories. In these cases, it has for example been further possible to extract information from single trajectories about transient spatial and temporal confinement [3], [11]. For the fluorescent dyes or proteins, another limitation in single molecule imaging is that the extension towards multi-color imaging requires multiple excitation lasers, and that simultaneous detection is challenged by the spectral overlap of the dyes used, which is usually significant when using more than two dyes.

More recently, quantum dots (QDs) have been introduced as an SPT probe [12]. QDs, which are fluorescent nanometer-sized semiconductor crystals, have unique optical properties [13]–[16], and are a very attractive compromise between gold particles and fluorescent dyes and proteins. In particular, QDs are distinguished by: a) their very large absorptivities and high fluorescence quantum yields, which render them exceptionally bright and allow tracking at frame rates close to 2 kHz [17]; b) their photo-chemical stability which enables imaging over extended time periods [18]; and c) their narrow, tunable emission spectra and overlapping excitation spectra which enable multi-color applications.

Biocompatible and biofunctional QDs are commercially available, or are easily prepared by chemical coupling employing standard chemistries [13], [19]–[21]. Biofunctionalized QDs are also intermediate in size between gold particles and fluorescent dyes and proteins [1]. These features make QDs ideal for SPT experiments for tracking of membrane species in the extracellular leaflet of the plasma membrane.

We and others have shown that it is possible to perform multi-color SPT of the same molecular species by use of QDs [22]–[27]. However, the potential of QDs has not yet been fully exploited for multi-color SPT as thus far no study of simultaneous tracking of different molecular species has been demonstrated. In this work, we have therefore set out to further extend our previous work of using a simple wide-field fluorescence microscope for multi-color SPT [24] to demonstrate that the technique can also be used for multiple colors of QDs having bio-specificity towards three distinct membrane species; a lipid (G_M1_), a GPI-anchored protein (CD59), and a transmembrane protein (EGFR). Further, we have validated that the resulting single QD trajectories are long enough to allow for robust single trajectory analysis.

## Materials and Methods

### QD Conjugations

#### Cholera toxin subunit B (CTB)-QD705

CTB-QD705 (peak emission at 705 nm) were custom made as previously described [28] from Qdot 705 ITK-carboxyl QDs (Invitrogen) and CTB (Sigma) via the cross-linker 1-ethyl-3(3-dimethylamino propyl) carbodiimide HCl (EDC) (Pierce). The CTB-QD705s used in this study were gel separated to ensure one CTB per QD.

#### CoEnzyme A (CoA)-QD655

CoA-QD655 were custom made from Qdot 655 ITK amino (PEG) QDs (Invitrogen) and CoA-SH (Covalys) via the cross-linker succinimidyl-4-(N-maleinidomethyl)-cyclohexane-1-carboxylate (SMCC) (Sigma) with slight variations from a previously published protocol [21] (Figure S1). The 655 QDs have a CdSe core and a ZnS shell, which is coated with an amino-pegylated triblock co-polymer [29]. A solution of amino QD655 (50 µL, 4 µM) in 50 mM borate buffer was activated with SMCC (5.6 µL, 10 mM) in DMSO for 1 h at RT (20°C). Unreacted SMCC was removed by size exclusion chromatography on a 50 mM MES buffer (2 mM EDTA, pH 6.0) equilibrated NAP-5 column by collecting the first 0.5 mL of colored solution. CoA-SH in PBS (10 mM phosphate, 138 mM NaCl 2.7 mM KCl, pH 7.2) was mixed with the activated QDs in molar ratios of 10 CoA pr. QD (1 µL, 2 mM) and 20 CoA pr. QD (2 µL, 2 mM) as compared to the original QD concentration and allowed to react for 1 h at RT. The reaction was quenched by addition of β-ME (10 µL, 10 mM) in milliQ-water for ½ h at RT. The CoA-conjugates (CoA-QD655) were purified by centrifugation in 50 kDa ultra-filtration tubes, and the buffer was exchanged to PBS on a Sephacryl 200 column. To characterize the effectiveness of the conjugation reaction and monodispersity of both the original amino, SMCC and CoA-QD preparations, we used agarose gel electrophoresis (Figure S1). Only the QDs made using 10 CoA per QD were used in this work.

### Cell Culture

Mouse embryonic fibroblasts from an Ink4a/Arf null mouse (IA32) were used for microscopy studies [24], [30], [31]. These cells are very large and flat making them highly suitable for SPT measurements in 2D. Cells were grown in humidified atmosphere at 37°C in 5% CO_2_. Cells were grown until 80–90% of confluence and split every third day in 1∶5–1∶10 ratios using the endopeptidase Trypsin (Sigma). Cells were grown in Dulbecco’s modified eagle’s medium (DMEM) with high glucose (Dulbecco), with standard concentrations of Glutamax (Gibco), penicillin-streptomycin (Sigma), and 10% fetal bovine serum (FBS) (Sigma). Cells were seeded at appropriate density and number (30,000) on coverslips in 6-well plates, and left for six-eight hours to attach to the glass. Cells were then transfected, left over night in media containing 10 µM biotin (Sigma), then labeled and imaged over the following two days.

### Plasmids and Transfection

The plasmids used for transfection were; pAEMTX-ACPwt-GPI (Covalys) encoding the GPI-anchored membrane protein CD59 with an extracellular ACP-tag, pcDNA3-EGFR-BLAP, encoding the EGFR with a biotin ligase accepter peptide (BLAP)-tag in an extracellular domain [32], pDISPLAY-BirA-KDEL encoding bacterial biotin ligase with an ER anchor [33], and K-Ras2-YFP (ATCC plasmid 10089283) encoding the first 19 amino acids of the C-terminus of the plasma membrane protein K-Ras2 with a YFP-tag. Cells were transfected with a total of 3.1 µg of DNA per well in a 1∶1∶1∶0.1 ratio of the plasmids and a 1∶2 (w/v) ratio of the transfection agent JetPEI (Polyplus Transfection).

### Multi-color Labeling and Cholesterol Depletion

Cells were washed twice in complete media before successive labeling with the three kinds of QDs. Cells were labeled in CoA-QD655 labeling solution (300 µL complete medium, 10 mM MgCl_2_, 1 nM CoA-QD655, 0.4 µM ACP Synthase) for no longer than 15 minutes at RT to minimize cross-linking of target molecules. The cells were washed three times in PBS (1% BSA, 0.1 mg/mL MgCl_2_, 0.1 mg/mL CaCl_2_) and incubated with or without 3 mM methyl-β-cyclodextrin (mβCD) for 10 minutes at 37°C to deplete cholesterol from the cells [34]. Cells were then labeled with a combination of 200 pM CTB-QD705 and 1 nM streptavidin (SAV)-QD605 (300 µL, PBS, 1% BSA, 0.1 mg/mL MgCl_2_, 0.1 mg/mL CaCl_2_) for two minutes with the addition of biotin (100 µL, 1 mM) after 30 s – 1 min to block further binding of SAV-QD605. Cells were washed three times in PBS (1% BSA, 0.1 mg/mL MgCl_2_, 0.1 mg/mL CaCl_2_) and imaged within 1½ h in presence of 50 µM β-mercaptoethanol (β-ME; Sigma) in order to minimize QD intermittency and maximize single molecule trajectory lengths [35].

### Microscopy Setup

Imaging was done using an Olympus IX-81 inverted microscope as has been described previously [24]. Fluorescence images were acquired at 10 ms integration time using a 100 W Hg arc lamp, a 470/40 nm bandpass excitation filter at 150X magnification with a 1.45 NA objective (Olympus) focusing on apical membranes of the lamella of the cells. Detection of all colors was done simultaneously through a QuadView emission splitter (dichroic mirrors at 585, 630, and 690 nm, and emission bandpass filters 535/30, 605/20, 655/20, and empty position) and an Andor EMCCD camera at 25 Hz. The camera has a pixel size of 16 µm, such that the projected pixel size in our case was 107 nm. The spectral overlap of the QD605, QD655, and QD705s among the image channels is such that less than 5% of the QDs are detected in the wrong image channel [24]. Image acquisition was controlled by Andor IQ software and movies of 1200 frames (∼48 s) were recorded at RT. The signal-to-noise in the image channel of the YFP-KRas2 fusion protein under the chosen imaging conditions (10 ms camera integration, 25 Hz imaging rate) is very low on an image frame by frame basis. Hence we have so far used these images only to provide a detailed image of the footprint of the plasma membrane of each cell for the duration of the time lapse sequence by generation of a Sum Intensity Projection image in ImageJ.

### Image Analysis

Single acquired time-lapse sequences were analyzed by use of a Particle Tracker plug-in in ImageJ [36] as has been described previously [24]. This analysis generates a text file containing the positions of the detected QD particle positions in each image frame as well as linked trajectories describing the motion of individual QDs in time. In this analysis, a major limitation to the use of QDs in SPT is made apparent by the generation of a large number of short trajectories rather than a more desirable few very long continuous particle trajectories. In order to minimize the number of inaccurate particle linking events, this analysis was done with conservative particle linking criteria, typically corresponding to a particle link range of 4–5 image frames and a maximum allowed particle displacement of two pixels per image frame to avoid artificial cross-over of particles. In order to further analyze the particle motion, the data were post-processed using custom written Mathematica routines. This post-processing included further linking of particle trajectories using a coincidence search routine in time and space of all other trajectories; the routine specified a minimal trajectory length of >50 steps, a maximum separation in time with other trajectories of <100 image frames, a maximum separation in space of <(0.1×the actual number of image frames in between trajectories) pixels^2^. This additional linking routine, which was validated by visual inspection, primarily resulted in the linking of single QD trajectories that are effectively immobile or alternatively displayed a relatively low mobility whereas the blinking off event was short.

We next calculated the mean squared displacements for each single trajectory, *m*, that contained *n*>50 image frames, and for all possible time intervals, *n t_lag_,*

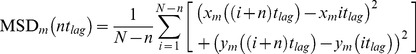
(1) where *t_lag_* is the time interval between images, and *N* is the total number of frames in a trajectory [37]. The MSD curves for each single trajectory, *m*, were curve fit at short time intervals, 1≤*n*≤5 (corresponding to 40≤n *t_lag_*≤200 ms) to a model for free diffusion:
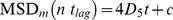
(2)where *D*_5_ is the diffusion coefficient and *c* is an off-set constant is related to the spatial precision by which we can determine the position of a single molecule [6]. For these fits, we weighed each data point used by the inverse of the variance (1/σ^2^).

### Fluorescence Correlation Spectroscopy (FCS) Measurements

The FCS measurements reported in this paper were made on a custom built multiphoton excitation microscope as has been described in detail previously [24]. For these measurements, we used a 60X, 1.2 NA water immersion objective. The excitation light source was a femtosecond Ti:Sa laser (Deep See, Spectra Physics, Mountain View, CA) and the excitation wavelength was 780 nm. The correlation data was collected at 50 kHz for ∼1 min where each reported measurement is the average measurement from at least five independent measurements. All measurements were performed in identical buffer conditions of 50 mM sodium borate pH 8.2 with 1% (w:v) BSA at room temperature (293 K). These measurements were calibrated by using Alexa488 labeled mouse IgG1 as a reference size standard with a known hydrodynamic radius, *R_H_*(Ms IgG1) = 5.6±0.2 nm [38] and by using the Stokes-Einstein relation. For measurements that were performed under identical buffer conditions, the relative size of a molecular species when compared to a size standard is given by:
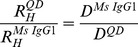
(3)


In order to also convert the relative hydrodynamic radius values to absolute values, we used the theoretical value for the solution viscosity of *η = *1.04 cP at 293 K [24], [39].

### Monte Carlo Simulations

The accuracy of the single particle trajectory analysis was evaluated by Monte Carlo simulations of free Brownian diffusion in 2D. In these simulations, we kept the total number of displacements of all trajectories constant at 10,000 while we varied the number of displacements per trajectory and in accordance the number of trajectories. Simulations were run for 10, 50, 100, 200, and 500 displacements per trajectory. The simulated diffusion coefficient, *D_Simulation_*, was 0.5 µm^2^/s and the time lag, *t_lag_*, was 40 ms in all simulations. The simulations were run in Mathematica by use of the RandomReal[] function for generation of two random numbers, a random direction of 0≤θ≤2π and a random displacement, *r*, where the distribution of the random displacements followed a Raleigh distribution, 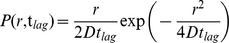. The simulated particle trajectories were subsequently analyzed in a similar manner to the experimental data by calculation of the MSD and by curve fitting. We next evaluated the robustness of the single trajectory data analysis by calculating the percentage difference for each trajectory between the simulated diffusion coefficient, *D_Simulation_*, and the fitted diffusion coefficient, *D_Fitted_*, from
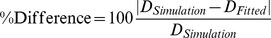
(4)and by determining the mean (± s.t.d.) percentage difference as a function of the number of displacements per trajectory. We further evaluated the accuracy by which we could recover the simulated diffusion coefficient, *D_Simulation_*, from curve fitting to the mean MSD(*n t_lag_*) curve, <MSD(*n t_lag_*)> for all displacements for each condition. In the subsequent sections we refer to the mean value of the %Difference as a measure of the accuracy of the single analysis method while we use the standard deviation of the %Difference as a measure of the precision.

### Statistical Tests

The non-parametric Kolmogorov-Smirnov test (K-S test) was used to evaluate the differences between the different populations of *D*_5_. The K-S test quantifies the distance between the cumulative density function of the two test populations. The null hypothesis is that the two test populations are drawn from the same distribution. The distributions are considered continuous, but are otherwise not restricted.

## Results

### Multi-species QD Labeling and Imaging

**Figure 1:**
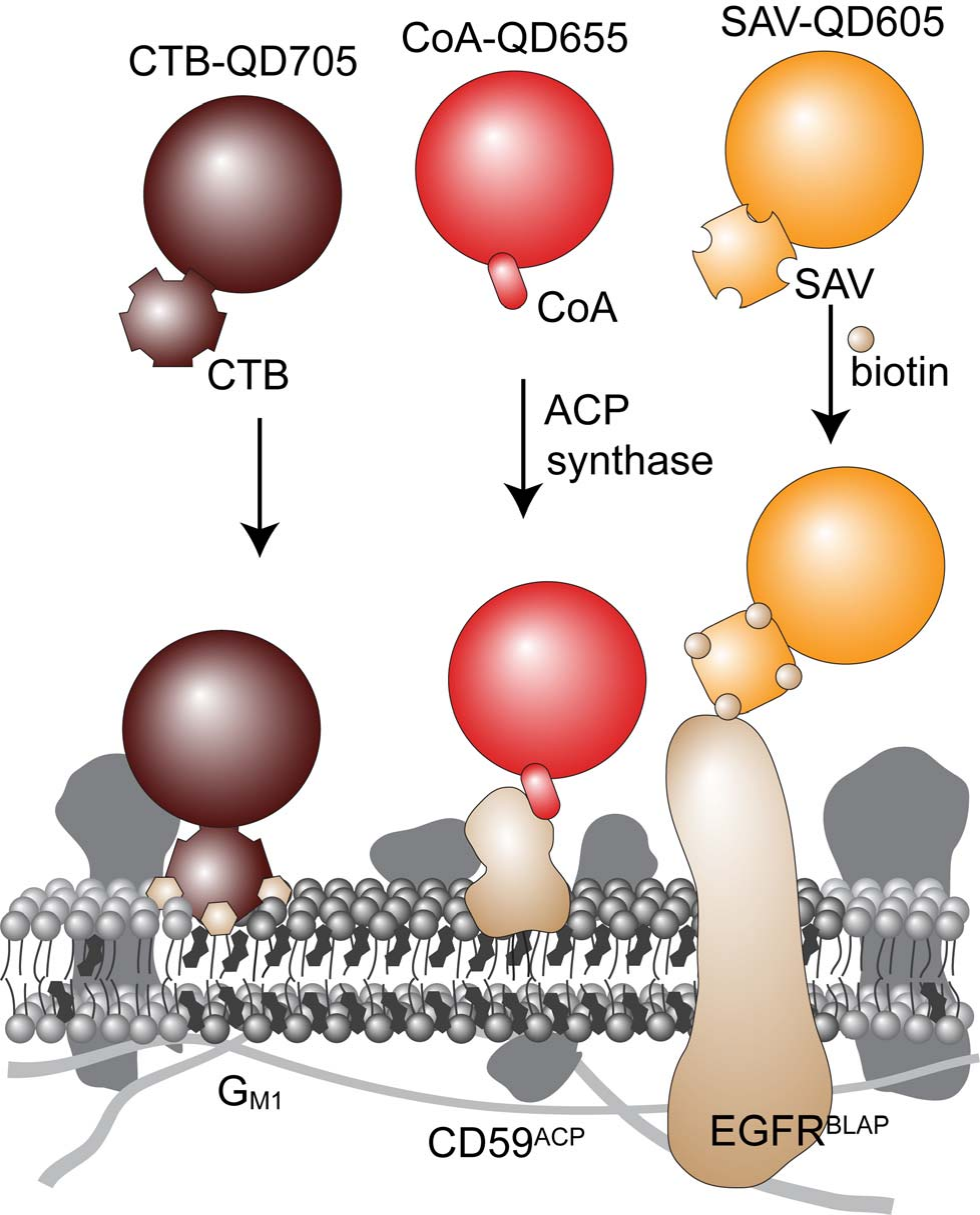
Multi-species SPT labeling strategy. CTB-QD705 labels specifically up to 5 ganglioside G_M1_molecules through direct binding of CTB to G_M1_ (left). CoA-QD655 is covalently coupled to GPI-anchored protein CD59^ACP^ in presence of the enzyme ACP synthase (center). SAV-QD605 targets biotinylated EGFR^BLAP^. The BLAP-tag is biotinylated during the secretory transport pathway when cells are grown in presence of biotin and are co-expressing bacterial biotin ligase (BirA-IgGκ-KDEL) [53].

Parallel multi-color SPT in live cells of the same species labeled with two [22], [25], [26], four [24], and very recently eight [27] different colors of QDs has been demonstrated. In this work, we show that it is also possible to extend multi-color SPT to include parallel imaging of different molecular species by targeting QDs of different colors to distinct membrane species using different targeting strategies. In this demonstration we have targeted; 1) a biotin ligase acceptor peptide (BLAP)-epitope tagged version of the epidermal growth factor receptor EGFR^BLAP^ [32], 2) an acyl carrier protein (ACP)-epitope tagged version of the GPI-anchored protein CD59^ACP^, and 3) ganglioside G_M1_ clusters (Figure 1), and followed their movement in the cellular plasma membrane over time. We have further treated cells with methyl-β-cyclodextrin to deplete cholesterol, and have investigated the effect of this treatment on the molecular movement.

The targeted molecules were in all cases labeled specifically immediately before imaging using respectively cholera toxin subunit B (CTB) conjugated QD705s (QDs with peak emission at 705 nm) for G_M1_ clusters [40], Coenzyme A (CoA) conjugated QD655s for CD59^ACP^, and streptavidin (SAV) conjugated QD605s for EGFR^BLAP^ [32] (Figure 1). We combined this three-color QD labeling with simultaneous imaging of a fusion protein consisting of the 19 C-terminal amino acids of K-Ras2 and yellow fluorescent protein (YFP). This fusion protein localizes to the plasma membrane with high specificity, and can therefore be used to obtain a high contrast image of the plasma membrane of each imaged cell. The four different membrane species were imaged simultaneously on a simple wide-field fluorescence microscope by single-color blue excitation, and by having a QuadView beam splitter with dichroic mirrors and color-filters matching the distinct emission spectra of the three QDs and YFP in front of the camera as previously demonstrated [24] and as described in Materials and Methods. The spectral overlap between the emission of the different QD colors was limited such that less than 5% of the QDs were detected in a wrong detection channel [24]. Furthermore, identical trajectories that appeared in more than one detection channel were deleted from further analysis hence precluding possible artifacts from spectral overlap. All multi-color time-lapse imaging experiments were performed at room temperature at an image acquisition frequency of 25 Hz (camera integration time = 10 ms, time-lag between frames was *t_lag_* = 40 ms). The spatial precision of the setup and at these acquisition settings was *δr* = (*δx* ^2^+*δy* ^2^)^1/2^ <30 nm [24].

**Figure 2:**
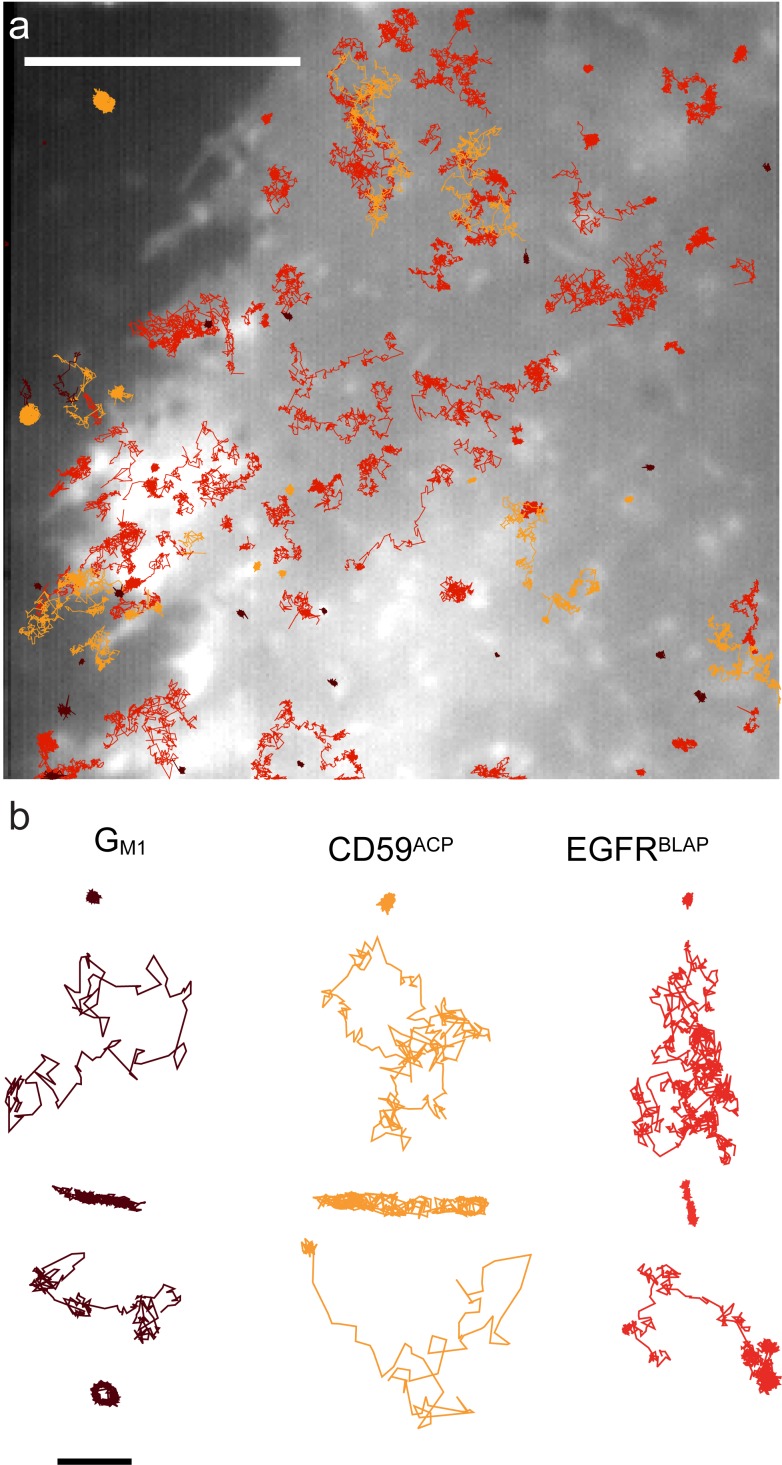
SPT trajectory examples. (**a**) Overlay of summed intensity image of K-Ras2-YFP and the calculated QD trajectories longer than 50 displacements. Dark red is G_M1_, red CD59^ACP^, and orange EGFR^BLAP^. Scale bar is 10** µ**m. (**b**) Examples of single trajectories for the three different molecular species. There are in all cases numerous examples of confined, free Brownian, and directed motion as well as various combinations thereof. Scale bar is 1** µ**m.

Single QD trajectories were reconstructed using the ImageJ plugin Particle Tracker [36] and custom written Mathematica routines as previously described [24]. A representative example of an overlay of the single molecule trajectories of the three QD tracked species on a K-Ras2-YFP membrane contrast image is shown in Figure 2a. It is clear that there was a very large heterogeneity among the observed motions of single molecules for all labeled membrane species with apparent examples of Brownian, confined, and directed motion and combinations thereof (Figure 2b).

### Single Trajectory Analysis for the Lateral Diffusion of G_M1_ Clusters, CD59 and EGFR

**Figure 3:**
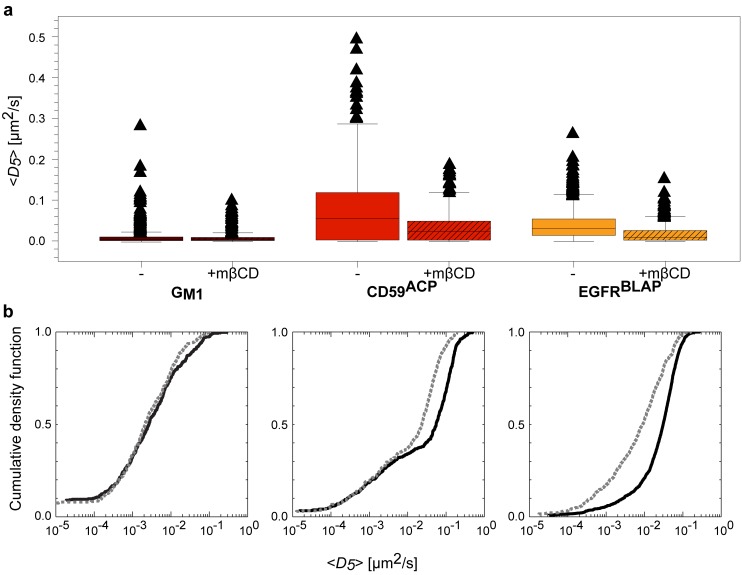
Single molecule diffusion of G_M1_, CD59^ACP^, and EGFR^BLAP^. (**a**) Box-and-whisker plots of the single molecule diffusion coefficients *D*_5_ for the three molecular species; G_M1_ (dark red), CD59^ACP^ (red), and EGFR^BLAP^ (orange), in non-treated (full color) and cholesterol depleted (hatched color) cells, respectively. (**b**) Cumulative density functions of the *D_5_* for untreated (black) and cholesterol depleted (grey dashed) cells. A statistical significant difference between the populations of *D*_5_ (K-S test) after cholesterol treatment was observed for CD59^ACP^ and EGFR^BLAP^. Statistical significant differences were also observed between the different molecular species both in non-treated and cholesterol depleted cells.

We calculated the mean squared displacement MSD(*t* = *n t_lag_*, Eq. 1) for each experimental trajectory with *N*>50 displacements and the results for the initial five displacements (1≤*n*≤5) were fitted to a Brownian diffusion model: MSD (*n t_lag_*) = 4 *D*_5_ *t* + c. This analysis gives a microscopic diffusion coefficient *D*_5_ that describes the lateral dynamics at short time intervals (40 ms<*t*<200 ms). The results of the single trajectory analysis are summarized in Table 1, and the distributions of *D*_5_ are shown in Figure 3.

**Table 1:** Trajectory and diffusion data.

	G_M1_		CD59^ACP^		EGFR^BLAP^	
QD conjugate	CTB-QD705		CoA-QD655		SAV-QD605	
Cell treatment	-	mβCD	-	mβCD	-	mβCD
# of trajectories	411	365	511	341	1557	499
<*N*> per trajectory	208	206	304	299	350	580
<*D_5_*>±s.e.m.[µm^2^/s]	0.013±6.8·10^-5^	0.008±4.1·10^-5^	0.074±16·10^-5^	0.034±11·10^-5^	0.038±2.1·10^-5^	0.018±4.7·10^-5^
MAD[µm^2^/s]	0.0025	0.0019	0.054	0.022	0.020	0.0081

Data from all single trajectories displayed in Figure 3. Data from non-treated cells was collected from a total of 18 cells, while data from cholesterol depleted (mβCD treatment) cells was collected from a total of 20 cells.

The distributions of *D*_5_ were found to be heterogeneous for all species in a significantly non-Gaussian way as determined from a Kolmogorov-Smirnov statistical test (K-S test). The K-S test was also applied in a pair-wise fashion comparing the same species with and without cholesterol depletion, and comparing the different species to evaluate whether the data – given the heterogeneity – suggested that the populations of *D*_5_ for the different cases were alike (p-values in Table 2). This showed a significant difference in lateral mobility between the different species independent of the cells being cholesterol-depleted or not. When comparing the same species before and after cholesterol depletion, a change between the populations of *D*_5_ was observed in the cases of CD59^ACP^ and EGFR^BLAP^, but there was no statistical significance difference for G_M1_ clusters (p-value = 0.17).

**Table 2:** p-values for Kolmogorov-Smirnov test.

Species 1	p-value	Species 2
*Intramolecular (+/−mβCD)*		
G_M1_ (−mβCD)	0.17	G_M1_ (+mβCD)
CD59^ACP^ (−mβCD)	9.4E-17	CD59^ACP^ (+mβCD)
EGFR^BLAP^ (−mβCD)	1.8E-42	EGFR^BLAP^ (+mβCD)
*Intermolecular (*−*mβCD)*		
G_M1_ (−mβCD)	1.3E-48	CD59^ACP^ (−mβCD)
CD59^ACP^ (−mβCD)	7.9E-30	EGFR^BLAP^ (−mβCD)
EGFR^BLAP^ (−mβCD)	4.1E-87	G_M1_ (−mβCD)
*Intermolecular (+mβCD)*		
G_M1_ (+mβCD)	1.4E-32	CD59^ACP^ (+mβCD)
CD59^ACP^ (+mβCD)	7.0E-11	EGFR^BLAP^ (+mβCD)
EGFR^BLAP^ (+mβCD)	5.9E-15	G_M1_ (+mβCD)

The table shows p-values for the significance of the difference in the populations of *D*_5_ between two species. All species compared are significantly different at significance level α = 0.05, except G_M1_ before and after cholesterol depletion (p-value 0.17).

To further quantify the molecular lateral heterogeneity in diffusion and the effect of cholesterol depletion, the relative change in the median absolute deviation (MAD) was calculated. The MAD was observed to decrease after cholesterol depletion by 25% for G_M1_ clusters, 59% for CD59^ACP^, and 59% for EGFR^BLAP^. Further, the population mean diffusion coefficient *<D*_5_ *>* was lowered for all three species by 38% for G_M1_ clusters, 55% for CD59^ACP^, and 52% for EGFR^BLAP^.

### Validation of Single Trajectory Analysis Approach

One of the major advantages of using QDs for SPT is that many of the resulting single trajectories typically consist of several tens to hundreds of displacements. In this study, we were able to detect hundreds of trajectories that consisted of more than 50 displacements for each condition. This allowed single trajectory analysis.

**Figure 4:**
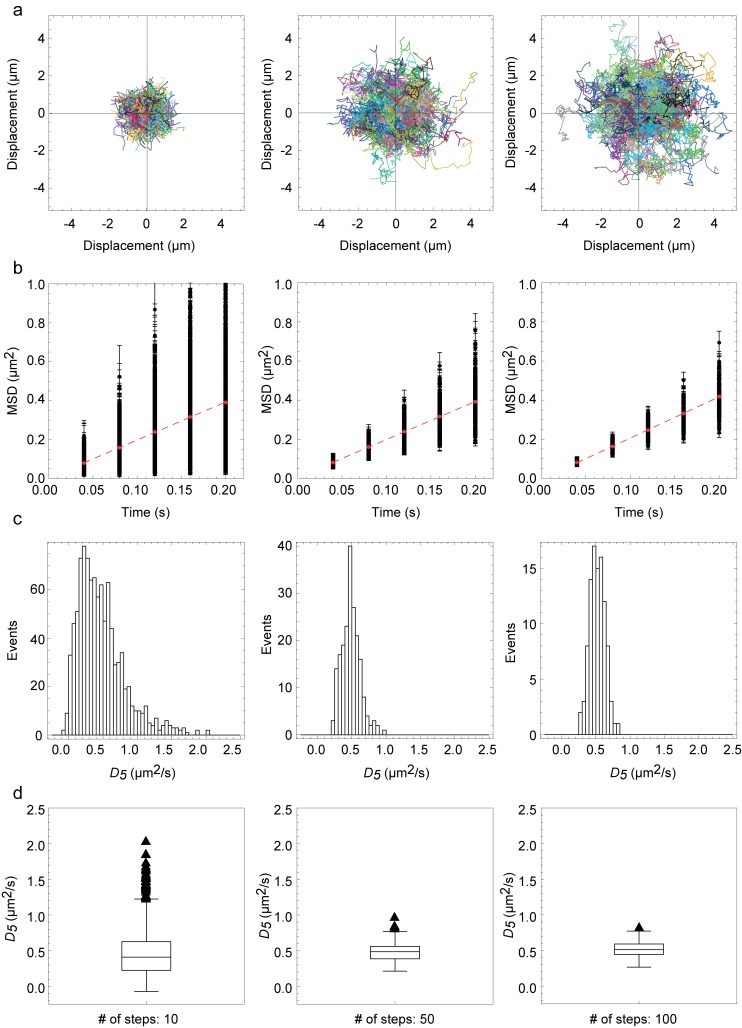
Monte Carlo simulations of 2D Brownian diffusion. The precision with which we could analyze single particle trajectories was estimated by a set of simulations. Left column: 1000 particle trajectories of 10 displacements per trajectory, Center column: 200 particle trajectories of 50 displacements per trajectory, and Right column: 100 particle trajectories of 100 displacements per trajectory. (**a**) All simulated particle trajectories (mixed colors). (**b**) MSD plots for each particle trajectory and best fit to the mean MSD(<MSD>) of all displacements (dashed red line). (**c**) Histograms of the fitted diffusion coefficients, *D_Fitted_*, for each independent simulated particle trajectory. (**d**) Box-and-whisker plots of *D_Fitted_* for each independent simulated particle trajectory. The numbers for fitted diffusion and error are given in Table 3.

In order to determine the robustness by which we could analyze single trajectories, we first performed a simple Monte Carlo simulation of Brownian diffusion in an infinite 2D plane. In this simulation, we evaluated the ability to recover a simulated diffusion coefficient from a mean squared displacement (MSD) analysis for the initial five displacements (1≤*n*≤5) as a function of the number of displacements, *N,* in the simulated trajectories (Figure 4).The complete results of the MSD analysis of the simulated data show that both the accuracy (as determined by calculating the mean percentage difference between the simulated diffusion coefficient, *D_Simulation_,* and the fitted diffusion coefficient, *D_Fitted_*) and the precision (as determined by the standard deviation of the accuracy) of the single trajectory analysis improves significantly as the length of the trajectories increases (Table 3).

**Table 3:** Results from Monte Carlo simulations.

Number of trajectories		1000	200	100	50	20
Number of displacements per trajectory		10	50	100	200	500
Single TrajectoryAnalysis	*D_Fitted_* (mean ± s.t.d.; µm^2^/s)	0.486±0.386	0.485±0.137	0.520±0.109	0.497±0.070	0.501±0.047
	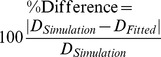	57±47	23±18	17±14	11±8.2	6.6±6.6
Average TrajectoryAnalysis	<*D_Fitted_*> ± a.s.e. (µm^2^/s)	0.504±0.001	0.502±0.001	0.489±0.001	0.497±0.001	0.502±0.001
	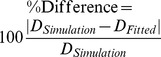	0.80±0.00	0.40±0.00	2.20±0.00	0.60±0.00	0.40±0.00

All simulations were done with *D_Simulation_* = 0.5 µm^2^/s and *t_lag_* = 40 ms.

The results of the simulations further validates that it is also possible to recover the magnitude of the simulated diffusion coefficient, *D_Simulation_*, with high accuracy for all possible simulated combinations either by analyzing single trajectories separately to determine the populations of *D*_5_ and determining the mean of these populations (Row 3 in Table 3) or by curve fitting to the mean <MSD(*t_lag_*)> (Row 5 in Table 3). This does, however, assume that the observed noise from the single trajectory SPT analysis is solely caused by the stochastic nature of Brownian diffusion. The observed extensive heterogeneity in the experimentally detected trajectories shown in Figure 2, however, suggests that this is not the case for our experimental data, but rather that the heterogeneity is a direct result of differences in mobility among different single particle trajectories.

Based on the results of these simulations, we selected a minimum threshold of *N* = 50 displacements for experimental trajectories that could robustly be analyzed by single trajectory analysis. With this threshold, the mean trajectory length of the experimental data was >200 displacements for all molecules (Table 3). The simulations showed a percentage error (±std.) of the fitted *D*_5_ compared to the simulated diffusion coefficient *D_Simulation_* of 23±18% for *N* = 50 displacements and 11±8% for *N* = 200 displacements. The corresponding percentage error for very short trajectories of *N* = 10 displacements was 57±47% thus confirming that single trajectory analysis is not possible for very short trajectories.

### Validation of QD Conjugates and Imaging Procedure

QDs have optical properties ideal for imaging at the single QD level and for multi-color applications, but their use is still questioned due to their size and multi-valency. Therefore, we performed an extensive series of control experiments in order to quantify the effect of the QD conjugates and the QD labeling procedure on each labeled molecule.

**Figure 5:**
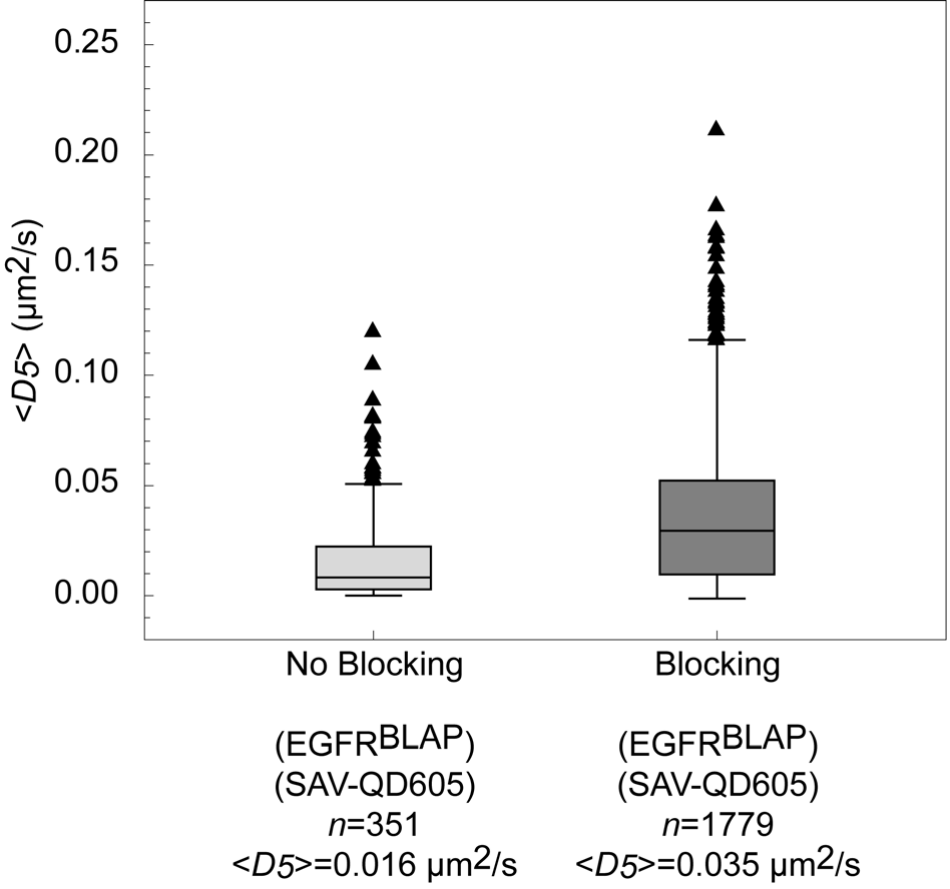
Effect of blocking SAV-QD605s with biotin. Box-and-whiskers plot of the diffusion coefficients for EGFR^BLAP^ targeted with SAV-QD605, with (dark gray) and without blocking (light gray) with excess biotin ½–1 min post SAV-QD605 labeling, respectively. The mean diffusion coefficient increased as indicated on the figure by ∼120%. The reduced mean diffusion coefficient when not blocking with biotin indicates that SAV-QD605 induced cross-binding of EGFR^BLAP^.

**Figure 6:**
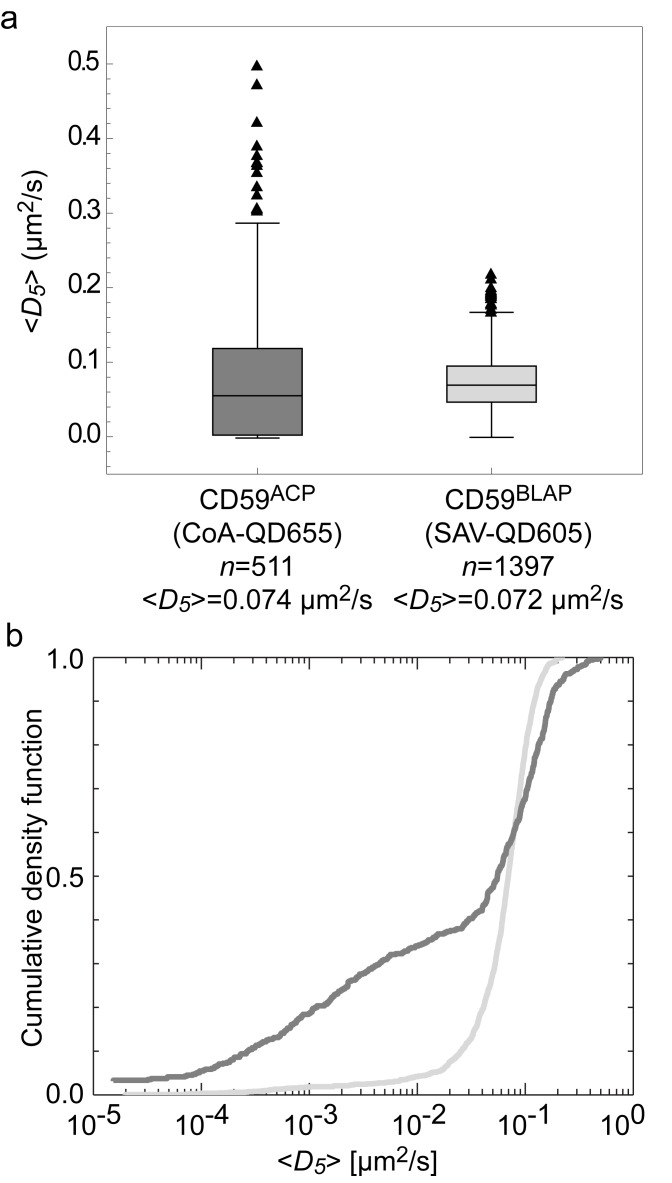
Comparison of SAV-QD605 and CoA-QD655 conjugates. (**a**) Box-and-whiskers plot of the diffusion coefficients for CD59^ACP^ and CD59^BLAP^ targeted with CoA-QD655 (dark gray) and SAV-QD605 (light gray). The mean diffusion coefficients of the two populations are numerically very close. (**b**) Cumulative density function plot of the diffusion coefficients of CD59^ACP^ and CD59^BLAP^showing that the distributions are non-identical.

The CTB-QD705 conjugates used were gel purified to enrich for QD conjugates bearing only one CTB [40]. However, CTB itself is pentavalent and it is therefore likely that small clusters of G_M1_ and not single G_M1_ molecules were tracked with CTB-QD705s. The low magnitudes of the diffusion coefficients for CTB-QD705s in this study further suggest that we are indeed observing the lateral motion of G_M1_ clusters. The monodispersity of the CoA-QD655 conjugates was checked by gel separation (Figure S1), and only conjugates having the lowest reaction ratio of CoA to QD, and also having high specific binding, were used (ratio 10∶1). The binding of CoA-QD655s to the plasma membrane ACP-target was further controlled by limiting the enzymatic incubation time with CoA Synthase. The commercial SAV-QD605s used are reported to have ∼15 SAV per QD [41]. Moreover, SAV is tetravalent and inherently there is therefore a high potential of cross-linking, when using these probes. Such cross-linking was minimized by addition of a >1000 fold excess of biotin shortly following QD labeling. This resulted in a 120% increase in the mean diffusion coefficient, *<D*_5_ *>*, as compared to labeling in the absence of the addition of excess free biotin (Figure 5). The specificity of the binding of the CoA and SAV QDs was tested by labeling non-expressing cells. Most such cells had no QDs on the surface, although some cells had a few QDs bound nonspecifically on their surfaces. A direct comparison of the CoA-QD655 and SAV-QD605 probes was made by targeting CD59^ACP^ and CD59^BLAP^, respectively (Figure 6). There was only a slight absolute difference in *<D*_5_ *>*; 0.074 µm^2^/s for CD59^ACP^ and 0.072 µm^2^/s for CD59^BLAP^. The distributions of the individual trajectory diffusion coefficients, *D*_5_, however, are not from similar populations, and therefore a direct comparison of the lateral dynamics is non-trivial. Finally, we also measured the hydrodynamic size of the three QD conjugates by fluorescence correlation spectroscopy (FCS) and found that the hydrodynamic radii (*R_H_*) of the different conjugates were approximately equal and were ∼10 nm (Table S1 and Figure S2).

**Figure 7:**
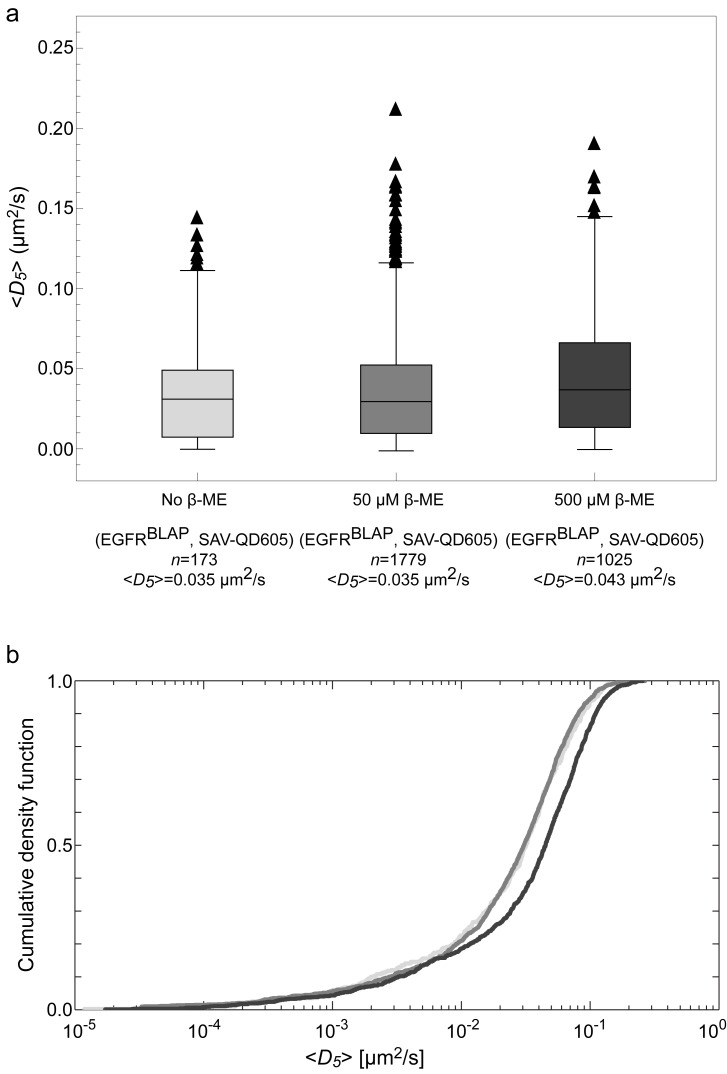
Effect of β-mercapthoethanol on SAV-QD605 trajectories. (**a**) Box-and-whiskers plot of the diffusion coefficients for EGFR^BLAP^ targeted with SAV-QD605, with no β-ME (light gray), 50 µM β-ME (medium gray), and 500 µM β-ME (dark gray) in the imaging buffer, respectively. The mean diffusion coefficients of the populations for no β-ME and 50 µM β-ME are identical, whereas there was an increase (23%) for 500 µM. (**b**) Cumulative density function of β-ME, 50 µM β-ME, and 500 µM β-ME, respectively. A K-S test showed no difference between the distributions when using no and 50 µM β-ME (p-value = 0.70), whereas there was a significant difference when using 500 µM β-ME (p-value<<0.05). In accordance with these results, all experiments were performed with 50 µM β-ME in order to reduce QD color shifting and blinking [35]. Addition of 50 µM β-ME increased the mean number of displacements per trajectory from 235 to 350 for 50 µM β-ME as compared to no β-ME.

In order to determine the effect of imaging in the presence of β-ME, we performed control experiments of EGFR^BLAP^ labeled with SAV-QD605s (Figure 7). These experiments showed that there was no quantitative difference in the diffusion between cells imaged with and without 50 µM β-ME, but that the mean trajectory length increased by ∼50% due to less blinking, as expected. Further experiments in the presence of 500 µM β-ME in contrast showed an increase in *<D*_5_ *>* by ∼40%. This suggests that steric hindrance due to e.g. disulfide bonds of the extracellular matrix could be an issue in the experiments, but that at least, low concentrations of β-ME do not affect the diffusion coefficient of EGFR^BLAP^. We believe that the use of such trace amounts of β-ME in this case is justified since stem cells are often grown in much higher concentrations to aid nutrient uptake [42].

## Discussion

In this study, we have extended our previous work in using a conventional wide-field fluorescence microscope for multi-color SPT with QDs [24] to simultaneously study the lateral dynamics of three distinct molecules, EGFR, CD59, and G_M1_ clusters, at the single QD level in live cells at frame rates of 25 Hz. We have accomplished this by using the optical properties of QDs and by design and preparation of spectrally separate colors of QDs for each molecule of interest. By this approach, we show that we are able to obtain sufficiently long (>50 displacements) single molecule trajectories for each labeled species to enable robust single trajectory analysis. This analysis confirms that the plasma membrane is a heterogeneous environment, and that the observed heterogeneity is above that expected from the random nature of Brownian diffusion. We further find that the distributions of diffusion coefficients differ between the different molecular species investigated. Finally, we find that cholesterol depletion using mβCD lowers the average diffusion coefficients and decreases the heterogeneity of the distributions of the diffusion coefficients for all three types of molecules.

The reported values of the mean diffusion coefficients for EGFR [43] and CD59 [44] are in agreement with previous studies carried out at similar temporal sampling rates in untreated cells, however the values for G_M1_ are 10–20 times slower than previously reported values from FRAP measurements [45]. Specifically, the values reported here for EGFR in untreated cells are similar in magnitude to previously published single molecule tracking values for EGFR that had been labeled with Alexa Fluor 546 conjugated Fab fragments [43] and is about five fold faster than EGFR clusters that had been labeled with Rhodamine conjugated EGF [46]. The values for CD59 in untreated cells are also similar in magnitude to previously published results for CD59 that had been labeled with Cy3 conjugated IgG but are about 3 fold slower than CD59 labeled with either Cy3 conjugated Fab fragments or Fab conjugated gold beads [44]. The values for G_M1_ in untreated cells are 10–20 times slower than previously reported values from FRAP measurements of fluorescently labeled CTB in COS7 cells with a 4.1 µm diameter laser bleach spot [45]. This occurred even though our CTB-QDs were gel purified to ensure one CTB per QD, as described previously [28]. However, the measurements reported here were done at a labeling concentration of 200 pM, which strongly favors the labeling of G_M1_ clusters, while the labeling concentration in the cited FRAP study was done at a saturating concentration of 1 µM, which strongly favors the labeling of single GM1 molecules [45]. Hence, we conclude that the measurements reported here are for G_M1_ clusters and not for single G_M1_ molecules. In addition, the mean diffusion coefficient in our measurements incorporates the motion of all detected molecules (as long as the detected single molecule trajectories were >50 displacements) while the mean diffusion coefficients from the FRAP measurements are solely derived from GM1 molecules that exchange over space scales that are equivalent to the size of the laser bleach spot. In the latter case, molecules that are spatially restricted during the measurement are contained within the immobile fraction and do hence not contribute to the reported measurements of the reported diffusion coefficients. Finally, the reported decrease in the magnitude of the mean diffusion coefficients upon cholesterol depletion with mβCD is consistent with most previous results [47].

The single trajectory analysis method which we used here was thoroughly validated by use of simulated data for Brownian diffusion in a 2D plane. Using this simple approach, we have demonstrated that the error in determining the diffusion coefficient from a single trajectory, at very short time intervals, 40 ms<*t*<200 ms, for a molecule that undergoes simple Brownian diffusion, is in the worst case scenario of our experimental data 20%, whereas the expected error for the average trajectory length of 200 displacements was approximately 10%. This sharply contrasts with results from the much shorter trajectories that can typically be obtained from single molecule studies using fluorescent dyes and proteins. As a result of photo-bleaching, such trajectories typically are much too short (median length of 5–15 displacements [6], [7]) to be precisely analyzed by single trajectory analysis (error close to 60%).

This study shows that QDs have many advantages in studying multiple different molecular species simultaneously at the single QD-labeled molecule level. In this study we have investigated only three different molecular species, but we have previously shown that our setup is also compatible with tracking four different colors of QDs, using QD565s as the fourth color [24]. Therefore, by applying QD565s bio-functionalized using a conjugation strategy different from than the ones that were used in this study e.g. the SNAP-tag system [48], antibody or antibody fragmented conjugated QDs [49], or ligand-conjugated QDs such as EGF-QDs [50], the method can easily be extended to study four different species. This study however also shows a major drawback of QDs that hampers a quantitative comparison of the different species studied: the valence of the QDs is difficult to control. In this work, we have optimized all the QD conjugates to achieve monovalent probes yet better and more readily available monovalent QD conjugates remain highly desirable [19], [26].

The great advantage of the multi-species approach described here is that it makes possible the simultaneous observation of up to four different molecular species. Thus, for example, it would be possible to investigate multiple membrane components during signaling cascade initiation. It has been shown that the diffusion coefficients of the same molecular species in the plasma membrane vary greatly on a cell-to-cell basis [51]. Our method enables the detailed comparison of variations within a single cell, or within the same local plasma membrane environment of a single cell, or within a single trajectory as has been recently done [27]. In addition, even though the low labeling density of the SPT techniques reduces the frequency of observed molecular interactions, a similar approach has been used to observe the formation of receptor dimers [27], [52].

## Supporting Information

Figure S1 **Synthesis of CoA-QD655 conjugates. (a)** In reaction 1 the NH_2_-group on the PEG-QD655 reacts with the NHS-ester of the cross-linker Succinimidyl-4-(*N*-maleimidomethyl)cyclohexane-1-carboxylate (SMCC). In reaction 2 the second reactive group of SMCC, the maleimide, reacts with the SH-group of SH-CoA. Reaction 2 is quenched in reaction 3 by the addition of excess β-ME which reacts and blocks unreacted maleimide. The final product is CoA-QD655. **(b)** 2% agarose gel. Lane 1: NH_2_-PEG-QDs. Lane 2: QDs activated with SMCC and quenched with β-ME. Lane 3: CoA-QD655 (molar ratio 10∶1). Lane 4: CoA-QD655 (molar ratio 20∶1). The QDs moved from negative to positive as indicated.
(TIF)

Figure S2 **Hydrodynamic radius of QD conjugates.** The hydrodynamic radii, *R_H_*, of the QD conjugates were determined by FCS as has been described previously [24]. Shown in a–d is the mean ± s.e.m. of *N* = 6 independent correlation curves for **(a)** Alexa488-labeled mouse IgG1, **(b)** CTB-QD705, **(c)** CoA-QD655, and **(d)** SAV-QD605. Also shown in a–d is the best fit to the theoretical expression of the mean of the autocorrelation curves, *G*(*τ*), for free diffusion in solution and using two-photon excitation. **(e)** Plot of the fitted diffusion coefficients in solution, *D_S_*, and the calculated hydrodynamic radius (*R_H_*, mean ± s.e.m.) from the Stokes-Einstein relation of the samples in a–d. All measurements were performed in 50 mM sodium borate pH 8.2 with 10 mg/ml BSA at RT and by using a Alexa488 labeled mouse IgG1 as a reference standard of a known hydrodynamic radius of *R_H_*(Ms IgG1) = 5.6±0.2 nm [38].
(TIF)

Table S1 **Hydrodynamic radius of QD conjugates.**
(DOCX)
