# Supplementary figures and images for: Simultaneous Multi-Species Tracking in Live Cells with Quantum Dot Conjugates
Source: PLoS One. 2014 Jun 3;9(6):e97671. doi: 10.1371/journal.pone.0097671 (PMC4043679; doi:10.1371/journal.pone.0097671)

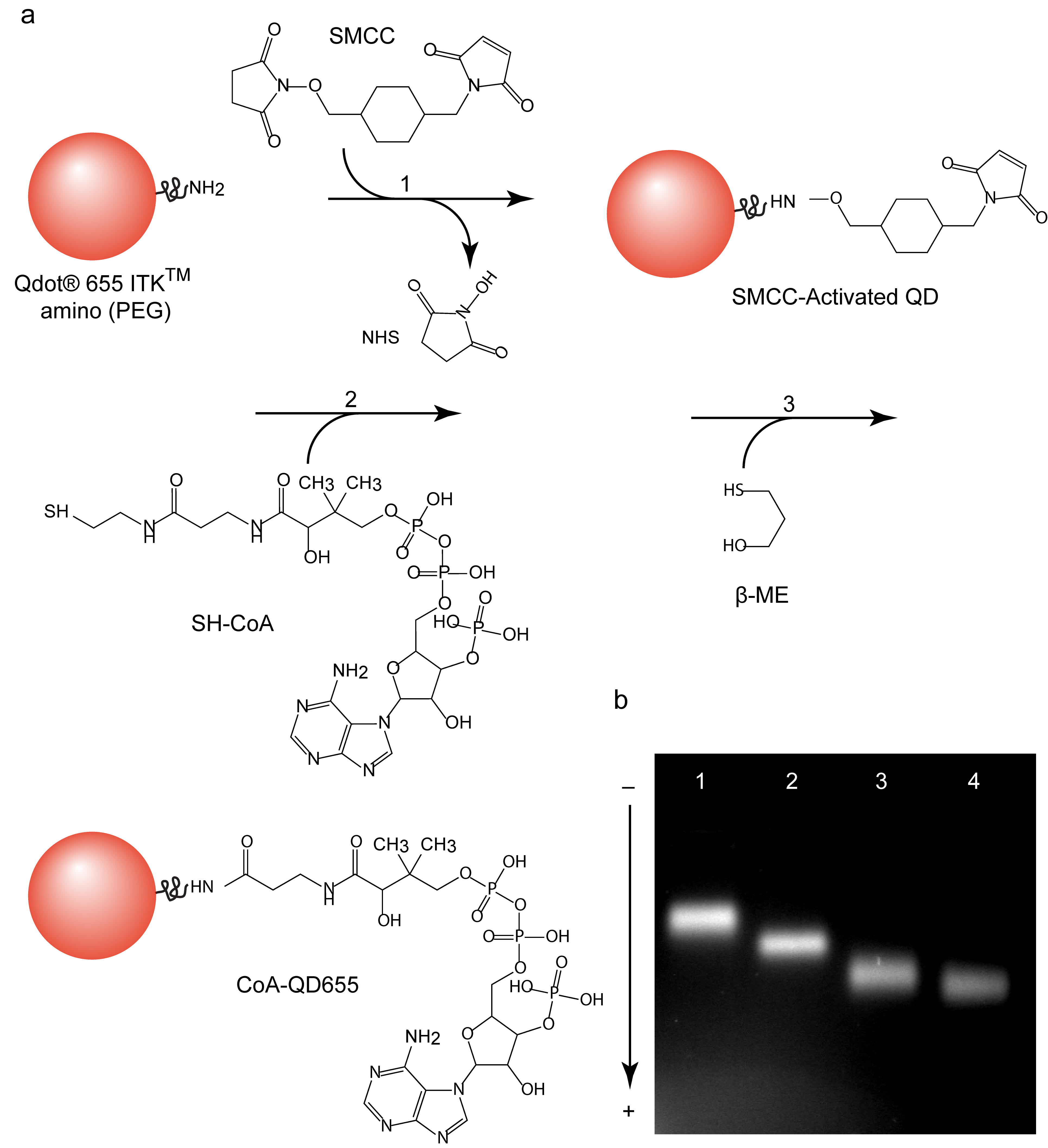

Supplement: Figure S1 — Synthesis of CoA-QD655 conjugates. (a) In reaction 1 the NH2-group on the PEG-QD655 reacts with the NHS-ester of the cross-linker Succinimidyl-4-(N-maleimidomethyl)cyclohexane-1-carboxylate (SMCC). In reaction 2 the second reactive group of SMCC, the maleimide, reacts with the SH-group of SH-CoA. Reaction 2 is quenched in reaction 3 by the addition of excess β-ME which reacts and blocks unreacted maleimide. The final product is CoA-QD655. (b) 2% agarose gel. Lane 1: NH2-PEG-QDs. Lane 2: QDs activated with SMCC and quenched with β-ME. Lane 3: CoA-QD655 (molar ratio 10∶1). Lane 4: CoA-QD655 (molar ratio 20∶1). The QDs moved from negative to positive as indicated. (TIF) [file pone.0097671.s001.tif]

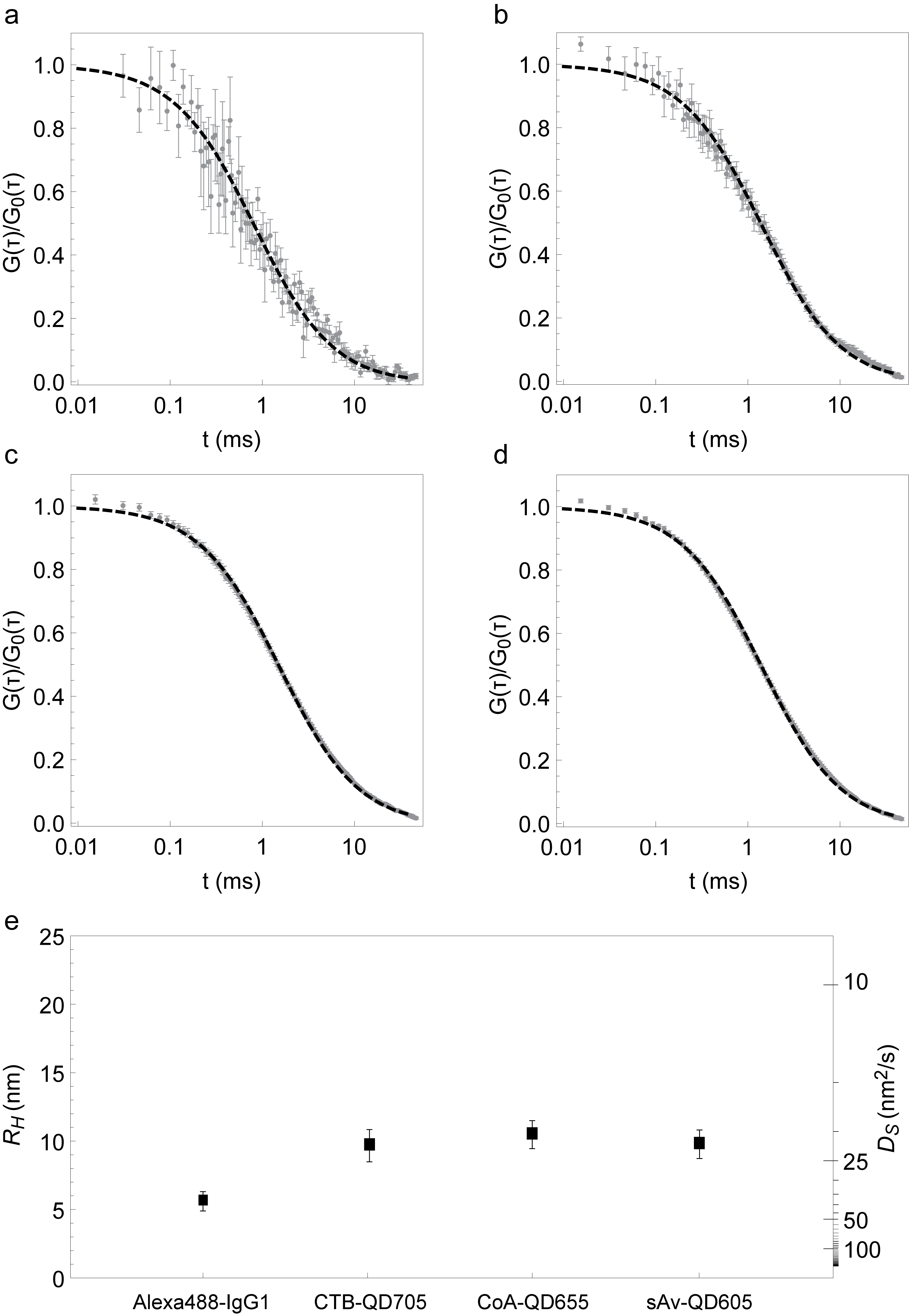

Supplement: Figure S2 — Hydrodynamic radius of QD conjugates. The hydrodynamic radii, RH, of the QD conjugates were determined by FCS as has been described previously [24]. Shown in a–d is the mean ± s.e.m. of N = 6 independent correlation curves for (a) Alexa488-labeled mouse IgG1, (b) CTB-QD705, (c) CoA-QD655, and (d) SAV-QD605. Also shown in a–d is the best fit to the theoretical expression of the mean of the autocorrelation curves, G(τ), for free diffusion in solution and using two-photon excitation. (e) Plot of the fitted diffusion coefficients in solution, DS, and the calculated hydrodynamic radius (RH, mean ± s.e.m.) from the Stokes-Einstein relation of the samples in a–d. All measurements were performed in 50 mM sodium borate pH 8.2 with 10 mg/ml BSA at RT and by using a Alexa488 labeled mouse IgG1 as a reference standard of a known hydrodynamic radius of RH(Ms IgG1) = 5.6±0.2 nm [38]. (TIF) [file pone.0097671.s002.tif]

**Table S1: Hydrodynamic radius of QD conjugates.**

| Probe | *N* | *DS* (μm2/s) | 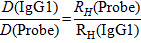 | *RH* |
| --- | --- | --- | --- | --- |
| Alexa488-Mouse IgG1 | 6 | 37.8±3.3 | 1.00±0.12 | 5.6±0.7 |
| CTB-QD705 | 6 | 21.9±1.7 | 1.73±0.20 | 9.7±1.2 |
| CoA-QD655 | 6 | 20.2±0.6 | 1.87±0.17 | 10.5±1.0 |
| SAV-QD605 | 6 | 21.7+1.1 | 1.75±0.18 | 9.8±1.0 |

Supplement: Table S1 — Hydrodynamic radius of QD conjugates. (DOCX) [file pone.0097671.s003.docx]
